# Acute Lymphoblastic Leukemia in an Adult After Renal Transplantation

**DOI:** 10.7759/cureus.27794

**Published:** 2022-08-08

**Authors:** Farhana Yaqoob Khan, Nabiha Zaman, Shahnila Latif

**Affiliations:** 1 Pathology, Methodist Hospital Specialty and Transplant, San Antonio, USA; 2 Medical School, University of North Texas Health Science Center, Fort Worth, USA

**Keywords:** solid organ transplant, acute lymphoblastic leukemia (all), b-cell acute lymphoblastic leukemia, high hyper-diploid, post-transplant malignancy, renal transplantation

## Abstract

Solid organ transplant patients are at an increased risk of developing various types of malignancies including hematological ones. The mechanisms behind these malignant changes are multifactorial. These include immunosuppressive agents, pre-transplantation cancer recurrence in the recipient, and de novo cancer development. Acute lymphoblastic leukemia is a rare malignancy in renal transplant recipients. Here, we describe the case of an adult male patient who underwent renal transplantation for end-stage renal disease due to diabetes and hypertension. He developed high hyper-diploid acute lymphoblastic leukemia four months after transplantation. This case is unique due to the presence of the high hyper-diploid cytogenetics of the B-cell acute lymphoblastic leukemia (B-ALL) occurrence in an adult renal transplant recipient.

## Introduction

Solid organ transplant recipients have an increased risk of developing cancer [1]. Several factors have been linked with the development of these cancers. These include immunosuppression, oncological viral infections (Epstein-Barr virus (EBV)), diminished immune surveillance of neoplastic cells, cancer already present in the transplanted tissue, and recurrence of cancer in the recipient [1]. The contributory effects of various immunosuppressive medications leading to the development of post-transplant cancer are not well understood.

The most common types of cancers that occur post-transplantation are skin cancers, non-Hodgkin’s lymphoma, lung cancer, liver cancer, and renal cancer [1]. Post-transplant lymphoproliferative disorders (PTLD) also commonly occur. This is a group of lymphoid cancers that occur in transplant recipients such as infectious mononucleosis PTLD, florid follicular hyperplasia PTLD, polymorphic PTLD, monomorphic PTLD, and classic Hodgkin-type lymphoma [2]. Acute lymphoblastic leukemia (ALL) is not part of the PTLD spectrum. ALL is a rare hematological malignancy amongst other hematological disorders seen in post-transplant patients [1,3]. Just 2.2% of calculated cases of ALL were observed in post-solid organ transplantation cases per 100,000 populations [1]. ALL is very common in children. High hyper-diploid (51-67 chromosomes) B-cell ALL (B-ALL) in adults is rare [4].

Here, we describe a case of a 32-year-old male who underwent cadaveric renal transplantation. He developed high hyper-diploid ALL four months post-transplantation.

## Case presentation

A 32-year-old Hispanic male presented to the emergency department with abdominal distension, discomfort, constipation, bloating, urinary frequency, and urinary hesitancy for the past five days. He was found to have ascites. He had a history of end-stage renal disease and was on peritoneal dialysis for the past three years on daily basis. He underwent a deceased donor pediatric en bloc renal transplant four months prior to presentation due to end-stage renal disease. His immunosuppression consisted of tacrolimus, mycophenolate mofetil, and induction therapy with Campath-1H (alemtuzumab). He had good allograft function and excellent urine output until he started having these symptoms. He had a history of diabetes since childhood and was on insulin therapy. His diabetes led to diabetic nephropathy, retinopathy, and neuropathy. In addition, he had obesity and hypertension stable on antihypertensives. His family history was unremarkable and he never used alcohol, illicit drugs, and smoking. He had a history of hepatitis C virus (HCV) treatment, and his viral markers were negative. His vital signs were normal. Apart from ascites and diffuse lymphadenopathy, no other significant findings were noted on physical examination upon presentation.

Routine hematological investigation showed pancytopenia with white blood cell (WBC) count of 1.2 K/UL, hemoglobin 12.5 g/dL, hematocrit 38.2%, mean corpuscular volume (MCV) 94.9 FL, red blood cell distribution width (RDW) 12.5%, platelets 121 K/UL, prothrombin time (PT) 14.4, and international normalized ratio (INR) 1.1. His creatinine was 2.2 mg/dL and blood urea nitrogen (BUN) 25 mg/dL. Peripheral blood smear showed normocytic anemia with occasional teardrop cells, significant leukopenia (neutrophil predominant), moderate thrombocytopenia with appropriate platelet morphology, and the circulating lymphocytes appeared immature and had no Auer rods.

Computerized tomography (CT) of the abdomen/pelvis confirmed ascites, diffuse lymphadenopathy, and splenomegaly. Paracentesis was performed and it was negative for malignant cells. His chest CT was normal. Lymph node excisional biopsy demonstrated partial involvement by B-ALL, but EBV-encoded RNA in-situ hybridization (EBER-ISH), acid-fast bacilli (AFB) stain, Grocott-Gomori's methenamine silver (GMS) stain, and Gram stains were negative for organisms. Immunophenotyping in the blastoid-appearing cells was negative for CD3, CD4, CD8, CD20, CD33, and positive for CD10, CD19, CD34, PAX5, and terminal deoxynucleotidyl transferase (TdT).

His polymerase chain reaction (PCR) was negative for cytomegalovirus (CMV) and EBV. Cytologic evaluation of the cerebrospinal fluid was negative for leukemic blasts. Histologic evaluation paired with immunohistochemical stains on the transjugular core biopsy of the liver tissue revealed intact and viable liver parenchyma containing expanded portal triads with an atypical infiltrate of TdT-positive B-lymphoblasts, consistent with porto-centric involvement by ALL.

**Figure 1:**
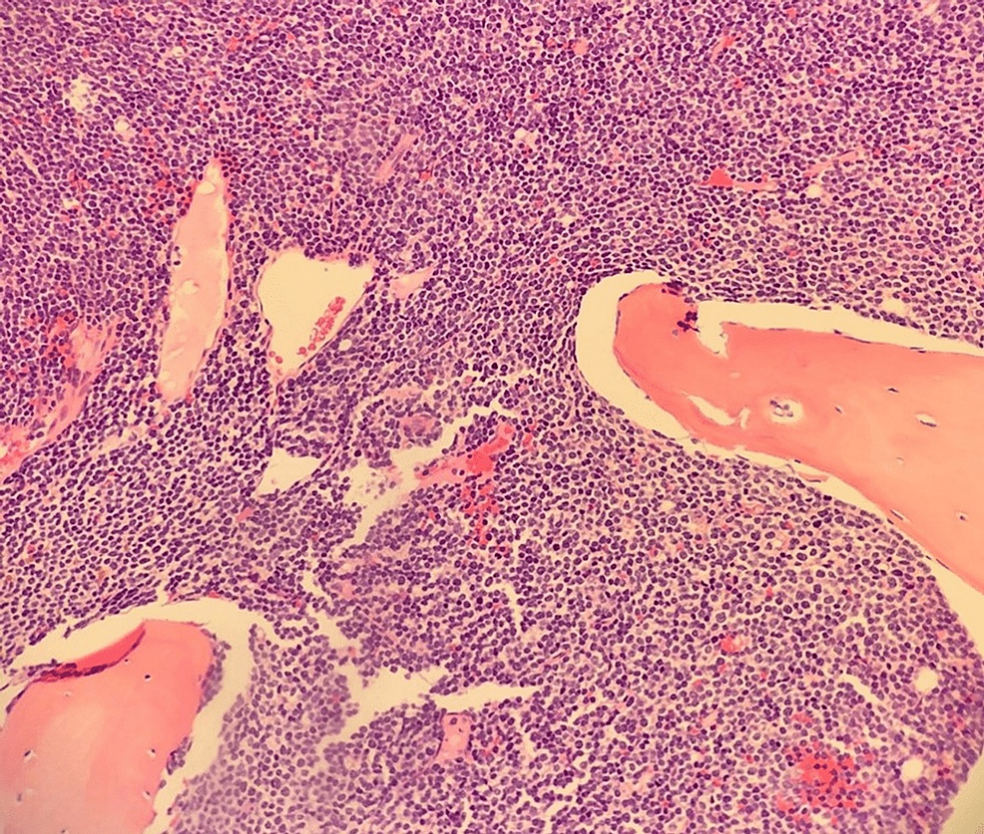
The figure depicts an H&E-stained section of the patient's marrow showing the marrow has been effaced by a monomorphic blastoid appearing population of small to medium-sized cells with round nuclear contours, scant basophilic cytoplasm, and occasional nucleoli with abundant mitotic activity. The remaining marrow elements are virtually absent H&E: hematoxylin and eosin staining

The patient’s marrow aspirate and biopsy showed hyper-cellular marrow with 100% cellularity effaced by B-ALL. The monomorphic population consisted of mononuclear cells with a blast-like morphology, scant basophilic cytoplasm, and absent Auer rods. The remainder of marrow elements, including granulocyte forms, were virtually absent with only rare erythroid precursors. Myeloid-to-erythroid (M:E) ratio is not relevant secondary to increased blast population. Bone marrow biopsy shows a complete replacement of the marrow elements by a monomorphic population of lymphoblasts of B-ALL (Figure 1).

Table 1 shows normal bone marrow differential count and patient's values.

**Table 1:** Bone marrow differential count. The first column shows types of cells found normally in the bone marrow with their normal percentages in the second column. The third column shows the patient's value of these cell types; his bone marrow comprises a monomorphic population of lymphoblasts replacing all other cell types, a characteristic of B-cell acute lymphoblastic leukemia

Cell type	Normal %	Patient’s values
Blasts	0-5	100% Lymphoblasts
Promyelocytes	1-8	-
Myelocytes	5-20	-
Metamyelocytes	13-32	-
Mature neutrophils	7-30	-
Mature eosinophils	1-6	-
Mature basophils	0-2	-
Monocytes	1-3	-
Lymphocytes	3-24	-
Plasma cells	1-3	-
Pronormoblasts	1-8	-
Normoblasts	6-32	-

**Figure 2:**
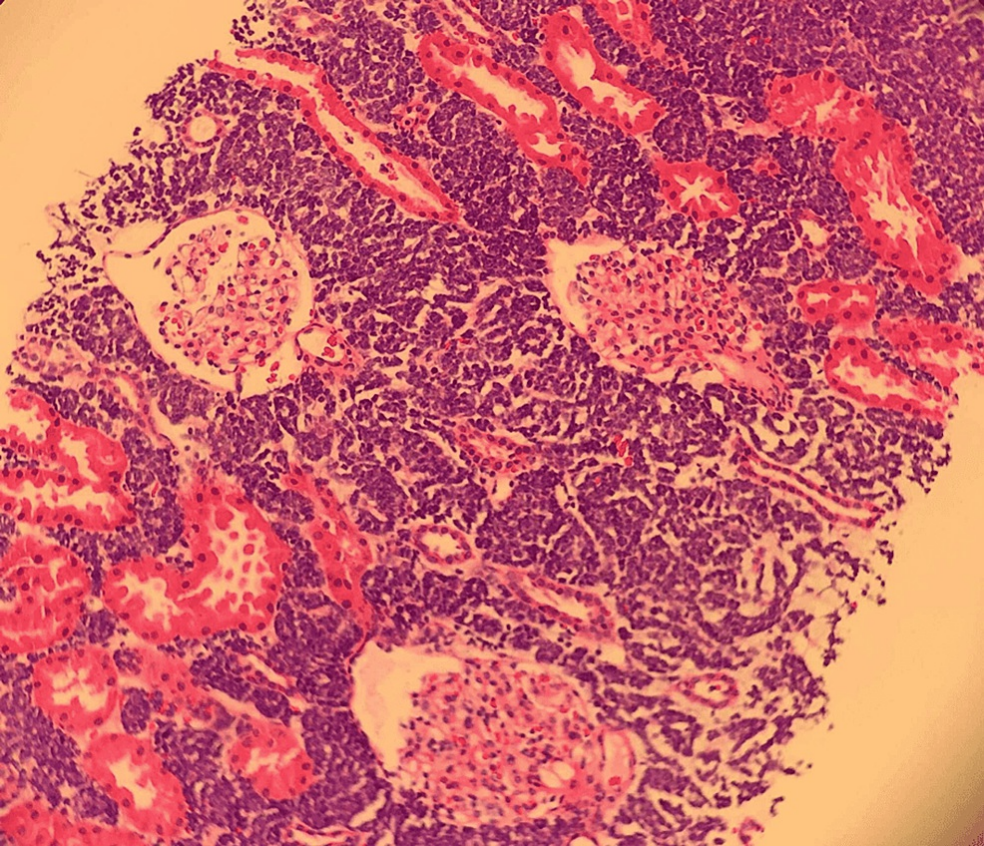
Transplant kidney biopsy showing lymphoblasts in the renal interstitium surrounding the glomeruli and tubules characteristics of B-cell acute lymphoblastic leukemia

Due to the patient’s history of renal transplantation, a kidney biopsy was performed. Needle core biopsy of the transplanted kidney showed interstitial infiltration by lymphoblasts, characteristic of diffuse B-ALL (Figure 2).

**Figure 3:**
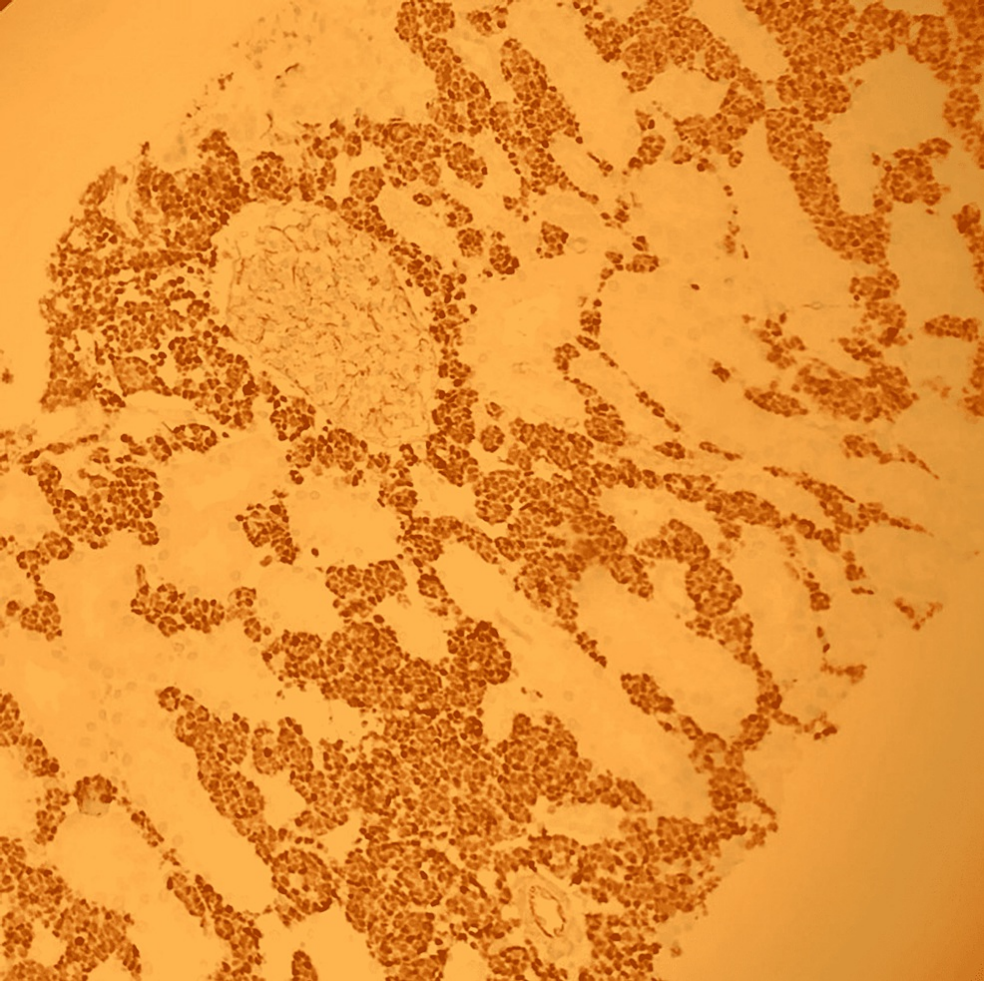
CD34 immunohistochemical staining of the transplanted kidney shows CD34 staining the lymphoblasts in the renal interstitium characteristics of B-cell acute lymphoblastic leukemia

The B-cells were strongly and diffusely positive for PAX5, TdT, and CD34. CD34 staining the tumor cells in the kidney is shown in Figure 3.

**Figure 4:**
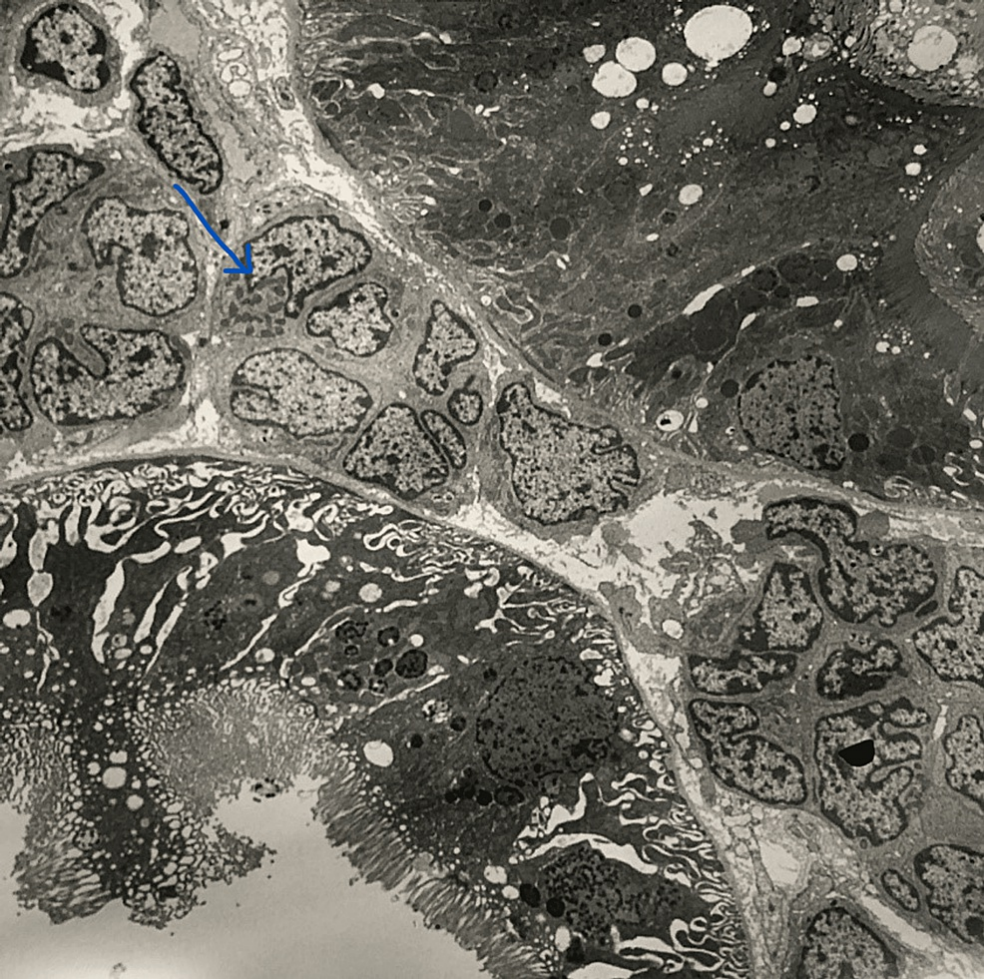
Electron microscopy of the transplanted kidney showing a lymphoblast (blue arrow) in the renal interstitium

No evidence of antibody-mediated or T-cell rejection was noted. Figure 4 shows electron microscopy of the renal interstitium invaded by the lymphoblasts.

**Figure 5:**
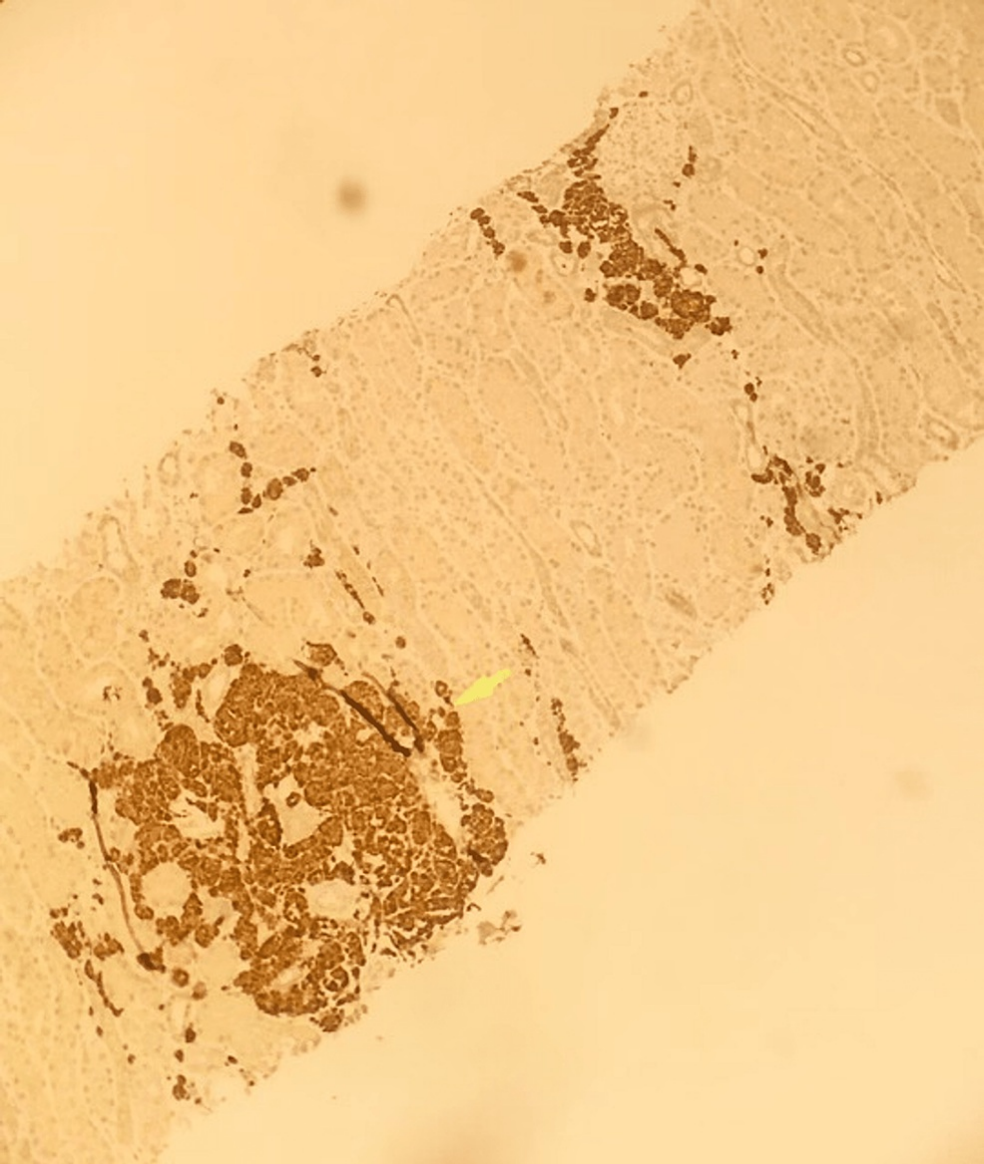
Immunohistochemical staining of the transplanted kidney showing positive TdT staining (yellow arrow) by the lymphoblasts TdT: terminal deoxynucleotidyl transferase

Immunohistochemical stains were positive for CD10, CD19, CD34, TdT, PAX5, BCL2, and negative for CD3, CD20, CD4, CD8, EBER ISH, BCL6, MUM1, CD30, CD43. CD33 was weakly positive. Figure 5 shows a positive TdT stain in the kidney.

**Figure 6:**
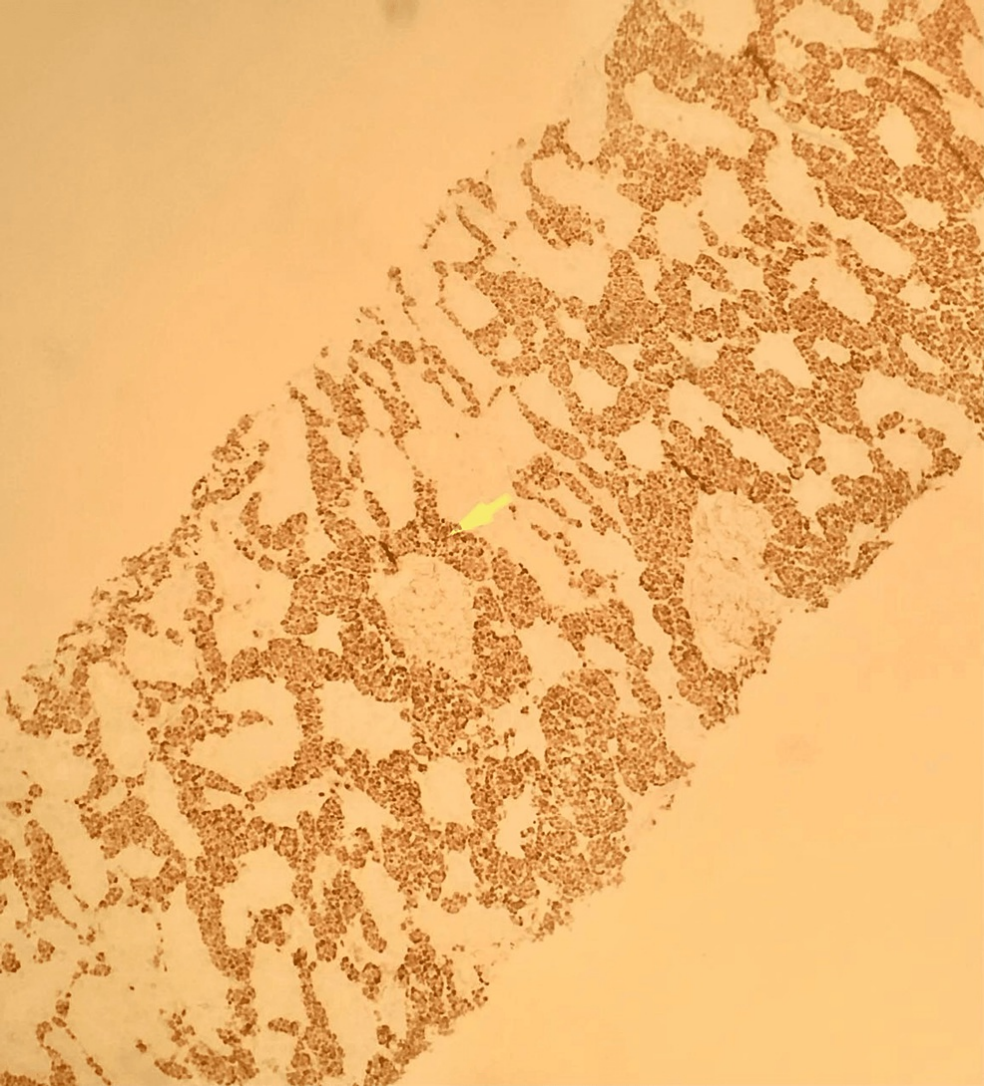
Immunohistochemical stain of kidney biopsy showing lymphoblast cells staining positive for PAX-5 characteristics of B-cell acute lymphoblastic leukemia

Figure 6 shows a positive PAX-5 stain in the kidney.

Since ALL is more common in children and rare post transplant, there was a high suspicion of a female child donor being the source of leukemia. Karyotyping performed on recipient marrow showed the following: 46, XY, add (9) (p13) [cp3]/53, idem, +2, +6, +21, +2 ~3m [cp2] /46, XY, which confirmed the source of ALL to be the recipient. Fluorescence in situ hybridization (FISH) showed trisomy of chromosome 6, tetrasomy of chromosome 21, trisomy of chromosomes 4, and deletion of the CDKN2A (p16) gene on chromosome 9 at 9p21. Extra copies of BCL2 and IGH fusion signal mean the presence of chromosome 18 and 14 abnormalities, respectively, were seen.

After discontinuing the immunosuppressive regimen, he underwent chemotherapy, and eventually lost his transplanted kidney.

## Discussion

Transplant recipients are at a high risk of developing cancer [1]. The use of immunosuppressive agents, cancer recurrence in the recipient, cancer in the donor tissue, and de novo cancer development are a few mechanisms of cancer development. Cancer is one of the leading causes of death in renal transplant recipients [5]. These malignant changes depend on the intensity and duration of immunosuppressive therapy, but we don't know the contributory effects of various immunosuppressive medications on the development of post-transplant cancer. The average time reported in the literature to develop leukemia after renal transplantation is 11.8 +/- 8.5 years [2,5]. This patient developed B-ALL four months after transplantation and immunosuppressive therapy. The cause of this rapid development of leukemia in this patient could be coincidental. The Hispanic population has a higher incidence of ALL because of polymorphisms in the ARID5B gene [6].

The most common types of cancers that occur after organ transplantation are non-Hodgkin’s lymphoma (14%), lung cancer (13%), liver cancer (9%), and renal cancer (7%) [1]. Post-transplant lymphoproliferative disorders (PTLD) are a group of lymphoid neoplasia that occur in solid organ transplant recipients. Lymphoproliferative malignancies developing after renal transplantation are usually of B-cell origin due to the involvement of oncogenic viruses. Leukemia is not part of these post-transplant lymphoproliferative disorders. The incidence of post-kidney transplant leukemia (PKTL) of all types has not been well established but is reported to be rare compared to solid organ cancers [3]. ALL is a rare malignancy after solid organ transplantation. There is only a 2.2% incidence per 100,000 person-years of ALL in solid organ transplantation cases [1].

High hyper-diploid B‐ALL is a type of ALL in which there is a gain of at least five additional chromosomes [4]. This type is a common cytogenetic abnormality in children and carries a favorable prognosis but in adults, this is the rarest, and its clinical significance is poorly understood [4]. High hyper-diploidy is present in only 7-8% of all adult B-ALL cases [4]. Limited information is available regarding the incidence of post-transplant high hyper-diploid B-ALL in the literature. The prognosis in adults is unfavorable compared to children due to more structural chromosomal abnormalities in adults [7]. These structural abnormalities can include dup(1q), del(6q), add(14q), and, infrequently, del(9p)/deletion of CDKN2A [7]. This patient had a del(9p)/deletion of CDKN2A, which is common in lymphoid neoplasms. Here, our patient had several aneuploidies such as trisomy 6, 21, and tetrasomy 4. Extra chromosomes 2 and 9 were also present. Gain of chromosomes 4, 6, 10, 14, 17, 18, 21, and X are most frequently reported in high hyper-diploid B-ALL cases [4]. The main chromosomal difference between adult and pediatric B-ALL with high hyper-diploidy is trisomy 6, which frequently occurs in adults but is uncommon in children.

## Conclusions

High hyper-diploid B-ALL is a rare type of leukemia in adults compared to children. This type of leukemia carries a poor prognosis in adults due to an increase in the number of structural chromosomal abnormalities.

One rare presentation of this type is in adult renal transplantation patients. The Hispanic male patient developed high hyper-diploid B-ALL four months after transplantation while on immunosuppressive therapy. We currently don't know the exact mechanisms behind this cancer development. So, more research is needed to find this type of presentation in other patients and populations. We also need to work on the complex genetic and environmental mechanisms to help the medical community understand this new presentation.
